# Antioxidant, Antitubercular and Cytotoxic Activities of *Piper imperiale*

**DOI:** 10.3390/molecules17044142

**Published:** 2012-04-05

**Authors:** Luis E. Diaz, Diego R. Munoz, Rosa E. Prieto, Sergio A. Cuervo, Diego L. Gonzalez, Juan D. Guzman, Sanjib Bhakta

**Affiliations:** 1 Engineering Faculty, University of La Sabana, A.A. 140122, Autopista North Km 7, Chía 11001, Colombia; 2 Mycobacteria Research Laboratory, Department of Biological Sciences, Institute of Structural and Molecular Biology, Birkbeck College, University of London, London, WC1E 7HX, UK

**Keywords:** *Piper imperiale*, phenolics, antioxidant, antitubercular, cytotoxicity

## Abstract

Phenolic compounds are widely distributed in Nature and act as pharmacologically active constituents in many herbal medicines. They have multiple biological properties, most notably antioxidant, antibacterial and cytotoxic activities. In the present study an attempt to correlate the phenolic composition of leaf, flower and wood extracts of *Piper imperiale*, with antioxidant, antitubercular and cytotoxic activities was undertaken. The total phenol content ranged from 1.98 to 6.94 mg GAE/gDW among ethanolic extracts, and gallic acid, catechin, epicatechin, ferulic acid, resveratrol and quercetin were identified and quantified by HPLC. DPPH and ABTS assays showed high antioxidant activity of the leaf extract (EC_50ABTS_ = 15.6 µg/mL, EC_50DPPH_ = 27.3 µg/mL) with EC_50_ in the same order of magnitude as the hydroxyquinone (EC_50ABTS_ = 10.2 µg/mL, EC_50DPPH_ = 15.7 µg/mL). The flower extract showed strong antimicrobial activity against *Mycobacterium tuberculosis* H_37_Rv. All the extracts exhibited dose-dependent cytotoxic effects against MCF-7 cancer cells. This is the first time that a *Piper* extract has been found to be highly active against *M. tuberculosis*. This study shows the biological potential of *Piper imperiale* extracts and gives way to bio-guided studies with well-defined biological activities.

## 1. Introduction

The *Piperaceae* family is one of the largest medicinally important plant families worldwide. *Piperaceae* species grow in tropical, warm climates distributed in eleven different genera (*Arcotonia*, *Macropiper*, *Manekia*, *Ottonia*, *Peperomia*, *Piper*, *Photomorphe*, *Sarcoscapum*, *Trianaeopiper*, *Verhuellia*, *Zipelia*) accounting for nearly 3,000 species [1]. The majority of species of the *Piper* genus occur in the tropical zone of America (700 spp.), followed by Southern Asia and Oceania (300 spp.) where the most economically important species, *Piper nigrum* (black pepper), historically originates [2]. In Colombia the *Piper* genus is widely distributed in Chocó, Antioquia, Valle and Cundinamarca, where a large diversity has been found to localize in the Sumapaz region [3]. Phytochemical reviews on the genus *Piper *have shown that it contains high amount of phenolic compounds, being the most important the flavonoid and lignan classes [4,5]. The hydroxyl groups of phenolics are of paramount importance for their biological activity as they can: (a) neutralize free radicals and donate hydrogen atoms or electrons [6]; (b) chelate metal ions in aqueous solutions [6,7,8], and (c) interact with proteins and cause protein precipitation [9,10]. It has been demonstrated that the antioxidant activity of phenolics offers multiple health benefits. For example, some phenolic compounds can reduce oxidative damage by limiting the amount of radicals generated by cellular metabolic activity [11]. Flavonoids have been found to be beneficial for cardiovascular disease because of their ability to adhere to monocytes in the inflammatory process of atherosclerosis [12,13]. Wine and its polyphenol constituents have the ability to reduce the susceptibility of low density lipoprotein to oxidation, increasing serum antioxidant capacity [14]. However not all beneficial effects of phenolics seems to be related to antioxidant activity. Phenols might exert effects within the gastrointestinal tract, for example by binding to iron [15] or they can interact with receptors and proteins such as the phytoestrogens [16] or they can prevent skin damage [17].

Phenolic components are essential for plants’ life, as they provide pigmentation to the flowers and fruits stimulating pollinators and seed dispersal. In addition they act as signal molecules in plant-plant interactions, they protect against damage caused by electromagnetic radiation and can also display defensive properties (antibacterial, antifungal) [18,19]. Recently, the interest in phenolics has been renewed because of their antioxidant, anti-inflammatory, anti-estrogenic, anti-mutagenic and anti-carcinogenic effects both *in vitro* and *in vivo *[20]. To our knowledge, only a few investigations have been carried out on *Piper imperiale *[21,22] and, as this species is traditionally used by local healers, it is necessary to link chemical compositions to biological effects. This study aims to investigate the total phenol content and the relation of the phenolic composition with antioxidant, antibacterial and cytotoxic activities of *P. imperiale*.

## 2. Results and Discussion

### 2.1. Total Phenolic Content

All extracts of *P. imperiale* displayed the presence of phenolic compounds, being the leaf extract the most abundant in phenols with a value of 6.94 mg GAE/g DW. Table 1 summarizes the value of the total phenol content in leaf, flower and wood extracts. The ranges of the amounts of phenols in the extracts varied from 6.94 to 1.98 mg of GAE/g DW. The wood extract had the lowest concentration of phenols (1.98 mg GAE/g DW), a value close to the amount found in the flowers (2.89 mg GAE/g DW), however the results had a statistically different significance (*p* < 0.05). The data indicates that the leaves which are more exposed to sunlight have higher amount of phenols, suggesting that they may play a role in extending the spectrum of absorption of light for making the photosynthetic process more efficient. This is in agreement with the observed fact that exposure to increased levels of UV radiation causes the leaves to redden, increasing the concentrations of phenols and flavonoids [23,24,25].

**Table 1:** Total phenolic content of leaf, flower and wood of *Piper imperiale*. The results are represented as mg of gallic acid equivalent per dried weight of plant.

*P. imperiale*	Phenols (mg GAE/g DW)
Leaf	6.94 ± 0.4
Flower	2.86 ± 0.2
Wood	1.98 ± 0.2

The correlation factor of the calibration curve was R^2^ = 0.9973, indicating good linearity. Values are mean ± SD of three samples of each extract, analyzed individually in triplicate.

### 2.2. Identification and Quantification of Phenolic Compounds by HPLC

**Figure 1:**
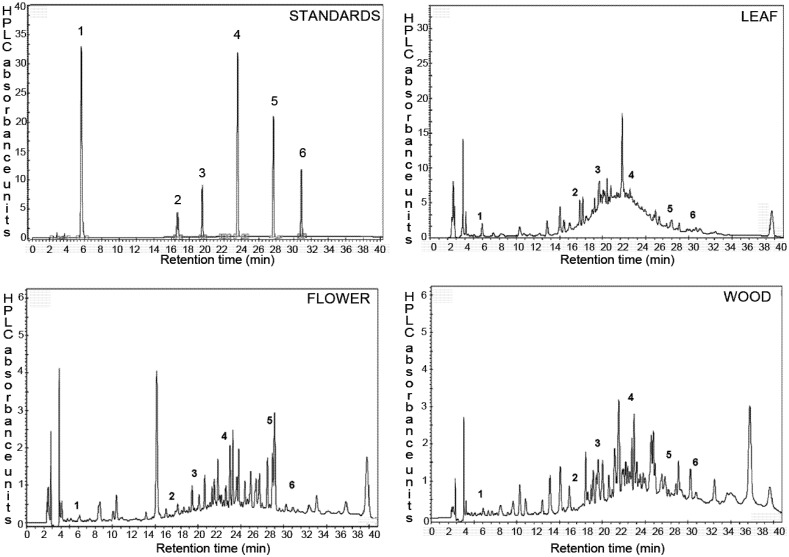
Chromatographic profiles at 280 nm of the phenolic components of the flowers, leaves and wood of *P. imperiale* and the phenolic standards.

The profile of phenolic compounds in extracts of leaf, flower and wood of *P. imperiale* was determined by RP-HPLC. The analysis method is a modification of the method used by de Quirós and collaborators [26], developed specifically for the analysis of phenolic compounds in natural extracts. The identification of individual compounds was carried out by comparing the retention times of signals in the samples with signals obtained for the standards (Figure 1). Six compounds were identified in all extracts according to their retention time and the HPLC extrapolated amount is shown in Table 2. Catechin and epicatechin are present in higher concentrations in the leaf, with 13 mg of catechin being present in 100 g of dried material. Epicatechin is also an abundant metabolite in all organs of these species, having values close to 2 mg/100 g of dried material. Flowers have the highest content of quercetin, and catechin is the most abundant secondary metabolite in the wood. It is interesting to note that the wood contains a high amount of ferulic acid, being the largest of the three extracts. The total amount of phenolic compounds analyzed by HPLC was 20.3, 6.3 and 4.7 mg/100 g DW for leaf, flower and wood respectively.

**Table 2:** Content (µg/100 g DW) of phenolic compounds in extracts of *P. imperiale.*

No.	Compound	Natural Product Class	Content in Extracts (µg/100 g DW)		
			Leaves	Flowers	Wood
1	Gallic acid	Phenolic acid	939.2 ± 74.2	13.1 ± 0.17	33.5 ± 0.43
2	Catechin	Flavanol	13613 ± 254	379.4 ± 15.4	457.1 ± 55.1
3	Epicatechin	Flavanol	2378 ± 41.7	1124 ± 5.30	1950 ± 17.7
4	Ferulic acid	Hydroxycinnamic acid	267.6 ± 8.48	75.0 ± 0.07	1629 ± 2.94
5	Resveratrol	Stilbene	1133 ± 28.3	1076 ± 201	97.8 ± 4.60
6	Quercetin	Flavonol	1954 ± 42.4	3661 ± 299	572.1 ± 16.8

Values are mean ± SD of three independent experiments.

A relation between the total phenol content measured by the Folin-Ciocalteu assay and the amount of phenols determined by HPLC was observed. The leaf extract had the highest amount of phenols by Folin-Ciocalteu and also had the highest integration values of HPLC peaks in comparison with the other extracts. Among the non-identified HPLC signals, two peaks of high intensity were observed in the chromatogram of the leaf extract having retention times of 22.3 and 39.6 min, which according to UV spectra and retention properties, can be hypothetically attributed to a flavanol and a low polarity stilbene, respectively. Major unidentified peaks were observed at 15.29 and 38 min in the flower and wood extracts which could not be attributed to any particular phenolic class.

Over 80% of the total amount of phenolic compounds found in the leaves and in the flowers of *P. imperiale* were found to be flavonoids, which have important biological properties such as antioxidant, antibacterial and cardio-protective [27,28,29]. Previous studies in species of the genus *Piper* have shown high levels of anti-inflammatory and antibacterial flavonoids in the leaves [30,31]. In the extracts of *P. imperiale*, catechin was found in high concentration and this flavonoid has received special attention as cytoprotective agent for the treatment of neurodegenerative diseases [32] or helping in wound healing [33]. Quercetin and epicatechin, also found in high concentrations in the extracts, have been shown to exert protection against cancer progression [34]. They also act as antioxidants *in vivo* by inhibiting pro-oxidant enzymes such as NADPH oxidases and lipoxygenases [35].

### 2.3. Evaluation of Antioxidant Activity

**Figure 2:**
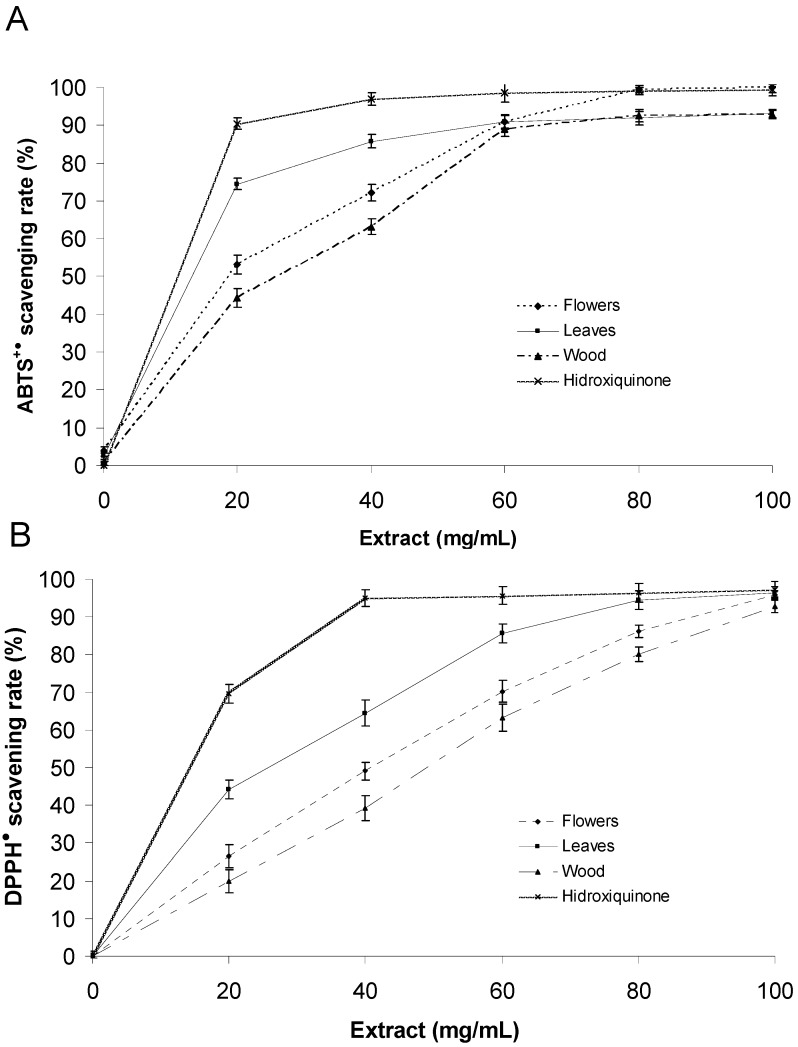
Percentage of scavenging activity using ABTS^+●^ and DPPH^●^ at different concentrations of the plant extracts (20, 40, 60, 80 and 100 µg/mL).

The extracts of *P. imperiale* showed interesting scavenging antioxidant properties (Figure 2). The highest antioxidant activity was found to be in the leaf extract (EC_50_ ABTS = 15.6 ± 0.8 µg/mL; EC_50_ DPPH = 27.3 ± 1.1 µg/mL) when compared to the flower and wood extracts (Table 3). The leaf extract had an EC_50_ in the same order of magnitude as the positive control hydroxyquinone (EC_50_ ABTS = 10.2 ± 0.6 µg/mL; EC_50_ DPPH = 15.7 ± 0.9 µg/mL). ABTS and DPPH assays gave comparable results for all the extracts and the control. The EC_50_ determined using the ABTS assay was consistently lower than the value determined using the DPPH assay. This trend has been found in other studies [36,37] and could be explained by the different time of end-point between the assays (6 min for ABTS and 30 min for DPPH). The reduction of DPPH and ABTS radicals was dose-dependent for all of the extracts tested.

**Table 3:** Free radical scavenging activity of the flower, leaf and wood extracts by the ABTS and DPPH assays. Hydroxyquinone was included as positive control.

Antioxidant activity	EC_50_ (µg/mL)			
	Flower	Leaf	Wood	Hydroxyquinone
ABTS	20.5 ± 1.2	15.6 ± 0.8	28.5 ± 0.9	10.2 ± 0.6
DPPH	39.7 ± 1.3	27.3 ± 1.1	47.8 ± 1.2	15.7 ± 0.9

Values are mean ± SD of three samples of each extract.

It is also evident that there is a correlation between the total phenol content measured by Folin-Ciocalteu/HPLC methods and the antioxidant capacity of the extracts of *P. imperiale*. In other words, the extracts with the highest amount of phenols were the extracts with the highest antioxidant capacity. However this correlation was not linear and therefore other non-phenolic compounds with antioxidant activity might exist in the extracts. These results are in agreement with the reported presence of carotenoids and vitamins in members of the *Piperaceae* family [38,39,40].

### 2.4. Anti-Tubercular Activity

Only the flower extract of *Piper imperiale* was able to inhibit the growth of *Mycobacterium tuberculosis* H_37_Rv (Table 4). The leaf and wood extracts were inactive even in the highest concentration tested (500 µg/mL). In contrast the flower extract was able to completely inhibit the growth of the bacilli at 75 µg/mL. The potent antimycobacterial effect of the flower extract was confirmed when the assay was performed against *Mycobacterium bovis* BCG Pasteur. Exactly the same MIC values were observed for the extracts against the vaccine BCG strain, demonstrating without doubt the antimycobacterial potential of this extract. Considering that it contains hundreds of low-concentration compounds as observed in the HPLC profile (Figure 1), it is remarkable such a low MIC, and suggest that highly potent anti-TB scaffolds might be present on it.

**Table 4:** Minimum inhibitory concentration (MIC) of the extracts of *Piper imperiale* on *Mycobacterium tuberculosis* H_37_Rv.

*Piper imperiale*	MIC (µg/mL) *Mycobacterium tuberculosis* H_37_Rv
Leaves	>500
Flowers	75
Wood	>500
Isoniazid	0.1

None of the phenolics identified in the extracts by HPLC has been reported to have any significant antimycobacterial activity. Catechin has been found to inhibit the growth of *M. tuberculosis* H_37_Rv at 200 µg/mL [41], however the MIC of the flower extract is much lower than this value, suggesting that other compounds might be responsible for the potent inhibitory activity of this extract.

### 2.5. Evaluation of Cytotoxicity of Natural Extract

**Figure 3:**
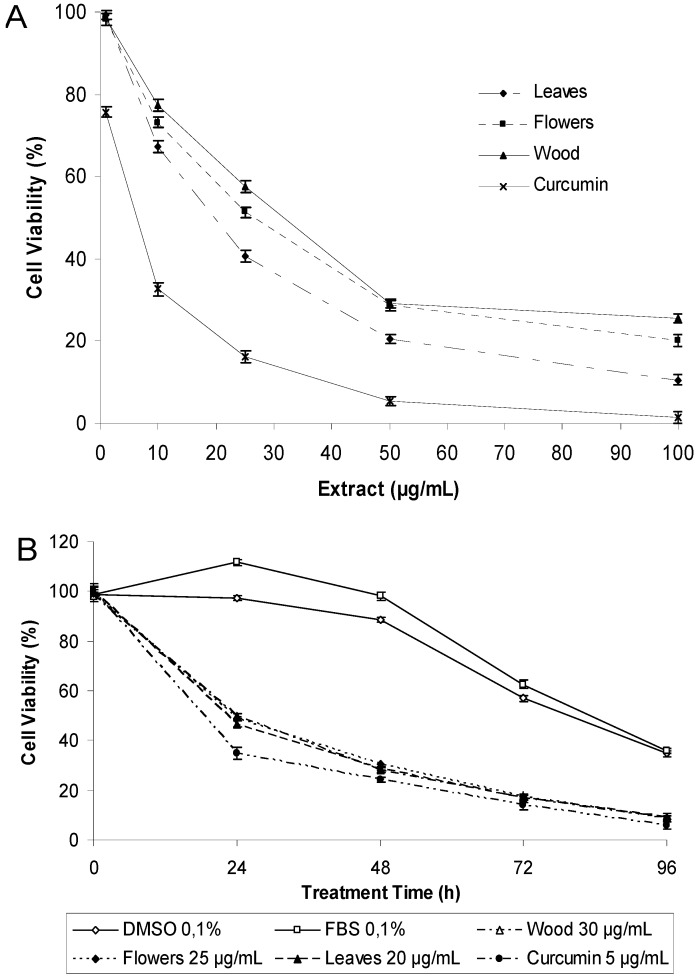
(**A**) Dose-dependent effect of curcumin and ethanolic extracts of *P. imperiale* in different concentrations (1, 10, 25, 50, 100 µg/mL) for 24 h at 37 °C on MCF-7 cell viability determined by MTT assay. Data shown represent the averages ± SD of three independent experiments. (**B**) Time-dependent effect of ethanolic extracts from the leaves, flowers and wood extract of *P. imperiale*. Data shown represent the means ± SD (n = 9).

A dose-dependent relation between the concentration of the extracts and the cytotoxicity against the cancer cell line MCF-7 was observed for all the extracts (Figure 3). All extracts demonstrated a comparable inhibitory activity. The leaf extract was the most active with an IC_50_ value of 18.6 µg/mL (Table 5). Curcumin was included as positive control as it is considered a potent molecule for cancer prevention [42,43,44], displaying an IC_50_ value of 8.2 µg/mL. To examine the time-dependent effect of curcumin or extracts of *P. imperiale* in MCF-7 cells, an assay using concentrations near the IC_50_ at 24, 48, 72 and 96 h was employed. Figure 3 shows that curcumin caused a remarkable anti-proliferative effect at a concentration two to three times lower than that of the extracts. It was clear that a spontaneous cell death process occurred after 24 h of treatment with extracts of *P. imperiale*. The cytotoxicity of the extracts was almost equal to that caused by curcumin after 72 h, and this effect was maintained through 96 h of treatment. The cytotoxic effect of the plant extracts was found to be time-dependent, however more than half of the cell death happening in the first 24 h.

**Table 5:** IC_50_ values of leaves, flowers and wood extracts of *P. imperiale* in tumor cells MCF-7 determined by MTT.

Extracts/Control	IC_50_ (µg/mL) *
Leaves	18.6 ± 1.2
Flowers	24.5 ± 1.5
Wood	30.7 ± 1.7
Curcumin	8.2 ± 0.7

* IC_50_ was calculated as the concentration (µg/mL) of extract causing a 50% inhibition of cell viability.

**Figure 4:**
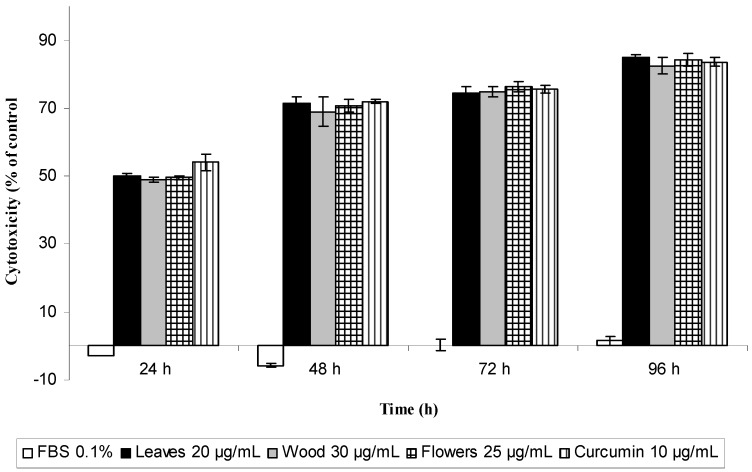
Cytotoxic effect of ethanolic extracts from the leaves, flowers and wood extract of *P. imperiale* determined by trypan blue exclusion method. The measurements were performed in triplicate in three different cultures. Data shown represent the means ± SD of three independent experiments. Significance differences with control (DMSO 0.1%) (*p* < 0.05).

The cytotoxity of the extracts was confirmed by the trypan blue exclusion method which gives a measure of cell mortality (Figure 4). A significant difference between control and extract-treated cells as well as positive control was observed along with a time-dependent increase in mortality. At 24 h, the cell mortality varied from 48.9% (wood 30 µg/mL) to 50.2% (leaves 20 µg/mL) while at 48 h it varied from 69.0% (wood 30 µg/mL) to 71.4% (leaves 20 µg/mL). At 24 h of treatment curcumin showed a stronger cytotoxic effect (54.1%) in comparison with the extracts, however at 48 h the effect was similar.

The results of trypan blue exclusion method are in agreement with the IC_50_ values calculated in the MTT assay. Both cytotoxic assays presented comparable results and the small differences observed in the cytotoxic values might be due to their assay principles, MTT reduction assessing the functional metabolic activity of mitochondria based on the enzymatic reduction of a tetrazolium salt by mitochondrial dehydrogenases of viable cells while trypan blue is based on cell membrane integrity. The use of different test confirms the cytotoxicity of the ethanol extracts from the aerial parts of *Piper imperiale*.

The cytotoxic activity of *Piper imperiale* is reported for the first time in this study. The data presented in this paper indicate that ethanol extracts are effective in suppressing the growth of MCF-7 cells in culture, as evidenced by a reduction in the cell density in a dose and time-dependent way. The growth inhibition was statistically significant starting from the time of 24 h of treatment for MCF-7 cells suggesting the existence of promising cytotoxic compounds in the ethanolic extracts. Even though the exact mechanism of action of extracts on MCF-7 cells remains unclear, it is noteworthy that this extract has cytotoxic and growth inhibitory effects. Our data clearly indicates that the ethanolic extracts of *P. imperiale* are cytotoxic and deserves further investigation in other cancer cell lines and tumor cells as a potential source of chemopreventive/chemotherapeutic agent for the treatment of cancer.

*Piper* species have been reported to have high phenolic content and antioxidant activity [41,45,46,47,48]. For instance, *P. guineense*, *P. nigrum*, *P. umbellatum* and *P. caninum* Blume have demonstrated antioxidant properties and high phenolic content. We cannot establish a direct relation between the results of cytotoxic activity and the antioxidant capacity of the phenolic compounds. However, although reports of *Piper* extracts displaying cytotoxic activity against tumor cells are few, most of them have mainly focused on amides or alkaloid-type compounds, which do not have significant antioxidant activity. Chabamide and chabamide G isolated from *P. chaba *Hunter showed potent cytotoxic activity against COLO-205 cell line, with IC_50_ values of 3.10 µg/mL and 0.018 µg/mL, respectively [49], and piperlongimin B isolated from *P. longum* inhibited cell proliferation of human leukemia HL-60 cell lines [50], but there is no mention of antioxidant activity. The results from this study suggest that *P. imperiale* cytotoxicity might be caused by phenolic compounds because the leaf extract which has the highest amount of phenolics causes cell death with the lowest concentration. Bio-guided isolation will tell us in due time whether this hypothesis is correct.

## 3. Experimental

### 3.1. Reagents

All reagents used in this study were HPLC or analytical grade. Acetonitrile, methanol and glacial acetic acid were purchased from Merck (Merck, Darmstadt, Germany). Phenolic standards (gallic acid, catechin, epicatechin, ferulic acid, resveratrol and quercetin), ABTS, DPPH, Folin-Ciocalteu’s phenol reagent, DMSO, hydroxyquinone and curcumin were obtained from Sigma (Sigma-Aldrich, St. Louis, MO, USA). Sodium bicarbonate, EDTA, PBS (phosphate buffer saline), Trypsin, DMEM (Dulbecco’s modified Eagle medium), and FBS (foetal bovine serum) were obtained from Gibco BRL (Pisley, Scotland, UK). Middlebrook 7H9, Middlebrook 7H10 agar mycobacterial growth media and OADC (oleic acid, albumin, dextrose, and catalase) and ADC supplements were purchased from BD Diagnostics (BD Diagnostics, Heidelberg, Germany).

### 3.2. Plant Material and Extracts

*P. imperiale* aerial parts were collected in March 2009 in Virolín, in the department of Santander, Colombia (6°3'56.7'' N/73°13'3.2'' W). A voucher specimen (COL516757) was deposited in the National Herbarium of Universidad Nacional de Colombia. The dried and powdered leaf (350 g), flower (148 g) and wood (547 g) material of *P. imperiale* were exhaustively extracted with 96% ethanol (500 mL) by maceration at room temperature (30 min). The solvent was removed in a rotary vacuum evaporator giving 28 g of leaf extract, 11 g of flower extract and 28 g of wood extract, respectively.

### 3.3. Total Phenolic Content

The modified Folin-Ciocalteu procedure [51] was used to measure the total phenolic content of each extract. Briefly, distilled water (10 mL), the plant extract (concentration) in 50 % aqueous methanol (1 mL) and Folin-Ciocalteu reagent (1 mL) were added to a 25 mL volumetric flask. After 5 min incubation in the dark, a 7% sodium carbonate aqueous solution (10 mL) was added and the volume was made up to 25 mL with distilled water. The solutions were mixed and allowed to stand in the dark at room temperature for 30 min. Absorbance was measured at 760 nm and the total phenol concentration was calculated from a calibration curve between 0 and 1.0 g/L of gallic acid (R^2^ = 0.998). The results are reported as mg of gallic acid equivalent per dried weight of plant (mg GAE/g DW).

### 3.4. HPLC Analysis

The chromatographic analysis was performed on a 1100 Agilent system (Agilent Technologies, Santa Clara, CA, USA) equipped with a quaternary pump, a degassing device, a 100 µL injection loop (Rheodyne, Rohnert Park, CA, USA) and a DAD detector. Chemstation software was used to control the conditions of HPLC. The separation was carried out at 28 °C on a 250 mm, 4.6 mm, 5 µm Luna C_18_ reverse-phase column (Phenomenex, Torrance, CA, USA). The following mobile phases were used in gradient programme: A (water-acetonitrile-acetic acid, 64:35:1 v/v/v) and B (water-acetic acid, 99:1 v/v). The following programme was used for the separation (the first value is the time in minutes and the second value is the percentage of mobile phase A): 0.0, 20; 4.0, 25; 8.0, 30; 12.0, 47; 16.0, 70; 20.0, 95; 22.0, 97; 24.0, 100; 35.0, 100; 37.0, 60; 40.0, 20, at a flow rate of 1.0 mL/min. The absorbance was recorded simultaneously at 280 nm and 360 nm. Approximately 120 mg of each extract was reconstituted in 1.0 mL of methanol. All the samples were filtered through a 0.45 µm membrane filter previous injection. The compounds were identified by comparing their retention times with the pure standards and confirmed by co-injection. Analyses were performed in triplicate.

### 3.5. DPPH Radical Scavenging Assay

The radical scavenging activity of the extracts was measured by a modification of the method of Hanato [52]. Five milliliters of a 0.2 mM DPPH methanolic solution were added to various concentrations (2, 4, 6, 8 and 10 g/L) of the plant extracts in methanol (50 µL) and a solution of DPPH (5 mL) and methanol (50 µL) was used as control. Hydroquinone was employed as a positive control. The mixture was shaken vigorously for 1 min and left to stand at room temperature for 30 min in dark. The absorbance was then measured at 517 nm and the scavenging capacity calculated as follows:


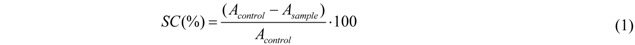


The concentration of extract needed for preventing half the oxidation of the DPPH (EC_50_) was calculated from the plot of percent of inhibition against the concentration of the extract. The assay was carried out in triplicate.

### 3.6. ABTS Radical Scavenging Test

Free radical scavenging activity of the extracts was determined using the ABTS radical cation (ABTS^+●^) decolorization assay [53]. ABTS was dissolved in water at 7 mM concentration. ABTS radical cation was produced by reacting ABTS stock solution with 2.45 mM potassium persulfate and allowing the mixture to stand in the dark at room temperature for 12–16 h before use. The ABTS^+●^ solution was diluted with ethanol to an absorbance of 0.70 at 734 nm and equilibrated at 30 °C. The blank reagent containing 20 µL of different concentration of the plant extracts in methanol (2, 4, 6, 8 and 10 g/L) was measured (A_0_) and after addition of 2.0 mL of diluted ABTS^+●^ solution, the absorbance reading was taken exactly 6 min after initial mixing (A_t_). Appropriate solvent blanks were run in each assay. All assays were carried out in triplicate.

### 3.7. Antitubercular Activity

The MIC determination against *Mycobacterium tuberculosis* H_37_Rv was performed using the spot culture growth inhibition assay (SPOTi) [54]. Briefly, the extracts were dissolved in DMSO at a concentration of 250 g/L. A solution at 2.5 g/L was also prepared from the concentrated stock. In a 24 well plate, different volumes of the extracts were added to the wells and completed with DMSO, maintaining a total volume of 4 µL. Thereafter molten Middlebrook 7H10 supplemented with 10% OADC (v/v) (2 mL) was added to each well, and the plates were allowed to dry overnight. A mid-exponential phase liquid culture (optical density at 600 nm between 0.6 to 0.9 absorbance units) of *Mycobacterium tuberculosis* H_37_Rv was diluted 1:100 with Middlebrook 7H9, and 2 µL was spotted into the middle of each well. After two weeks of incubation at 37 °C the plates were observed for growth. MIC values were determined as the minimum concentration of extract where there was no visible mycobacterial growth. The assay was also performed on *Mycobacterium bovis* BCG Pasteur strain.

### 3.8. Cytotoxicity of the Plant Extracts on MCF-7 Cell Line

The human breast cancer cell line MCF-7 was purchased from the American Type Culture Collection. The cells were cultured in a 25 cm^2^ cell culture flask on Dulbecco’s modified Eagle’s medium (DMEM) supplemented with 10% foetal bovine serum (FBS), 10 units of penicillin and 10 µg/mL of streptomycin, at 37 °C in a humidified incubator with controlled 5% CO_2_ supply. When the cells were around 90% confluent, they were detached through trypsinization using 0.25% trypsin-EDTA solution. The cells were counted using a haemocytometer and 10^4^ cells were seeded in each well on a 96-well sterile cell culture plate. After 24 h of incubation, the plant extracts were added at different concentrations (1, 10, 25, 50, 100 µg/mL). Curcumin was used as positive control in the same concentrations and DMSO was employed as negative control. After 24 h of exposure to the plant extracts, the cell viability was detected using 3-[4,5-dimethylthiazol-2-yl]-2,5-diphenyl-tetrazolium bromide (MTT) (Sigma-Aldrich, St. Louis, MO, USA). The cell monolayers were washed with phosphate buffer saline and 100 µL per well of filter-sterilized aqueous solution of MTT (5 mg/mL) were added to each well. The plates were then incubated at 37 °C for 4 h and then the absorbance recorded at 540 nm on a microplate reader. The percentage of cell viability was calculated in comparison with the DMSO treated experiment. In the time-dependent cytotoxic experiment, the cells were treated with leaf, flower or wood extracts at 20, 25 and 30 µg/mL respectively with different incubation times (24, 48, 72 and 96 h). Cell viability was determined with MTT colorimetric assay and measurements were done in duplicate in three different cultures. Curcumin (10 µg/mL) was used as positive control and DMSO (0.1%) was employed as negative control. Experimental values are given as means ± standard deviation.

Cellular cytotoxicity of *Piper imperiale* extracts was confirmed using trypan blue exclusion method using a hemocytometer [55]. Briefly, MCF-7 cells were seeded in 6-well plates at 10^6^ cells/well. The cells were cultured in DMEM supplemented with 0.1% FBS (foetal bovine serum) for 24 h at 37 °C. The cells were washed twice with PBS 1X (phosphate buffer solution, pH 7.4: 137 mM NaCl, 2.7 mM KCl, 8 mM Na_2_HPO_4_, 1.5 mM KH_2_PO_4_). Cells were exposed to the extracts at different concentrations (flower: 2.5 µg/mL, leaf: 20 µg/mL, wood: 30 µg/mL) and incubated for 24 and 48 h and then trypsinized using 0.25% trypsin–EDTA solution, centrifuged and re-suspended in 500 µL of PBS. Curcumin (10 µg/mL) was used as positive control at the same concentrations and DMSO (0.1%) was employed as negative control. Trypan blue 0.4% in PBS (100 µL) was added for 2 min to an equal volume of cell suspension. Four randomly chosen visual fields were examined in each sample and dead (stained) and viable (unstained) cells were counted (approximately 100 cells per observation field). The percentage of cytotoxicity was calculated as follows:


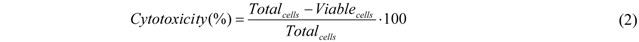


### 3.9. Statistical Analysis

Statistical analysis was performed using the two-tailed *t *test (Graph Pad software Inc, San Diego, CA, USA). Values were considered to be statistically significantly different when *p *values were less than 0.05. Each experiment was performed at least three times. Experimental values are given as means ± SD.

## 4. Conclusions

All the extracts of the aerial part of *P. imperiale* showed a considerable content of phenols, especially the leaves, which also showed the highest antioxidant activity in ABTS and DPPH assays and the highest cytotoxicity against MCF-7 cells. This effect can be attributed, at least partially, to its high content of flavonols and stilbenes. However it is still necessary to understand which specific compound is causing the cytotoxic effect and also what is the exact mechanism of action for MCF-7 cell killing. The flower extract of *P. imperiale* showed a potent antibacterial activity particularly against the pathogenic bacteria causing tuberculosis, and therefore this extract deserves attention as a source of antimycobacterials. These results suggests that *P. imperiale* ethanolic extracts should be considered as starting material for the bio-guided isolation of potentially important antioxidant, antibacterial and cytotoxic chemical scaffolds.

This study of the biological potential of *Piper imperiale* extracts is very important and timely as it pave the way for the bio-guided isolation of compounds with well-defined biological activities, but also it helps to bring the attention to the enormous potential of *Piper* species in biomedicine. This is the first time that the flower extract of *Piper* species has been found to be extremely active against *M. tuberculosis*. Also this is the first time that a relation between antioxidant activity and cytotoxicity has been hypothetized for a *Piper* species. These findings are of paramount interest for the phytochemist and natural product chemist, especially those working on *Piper* species.
